# C8ORF88: A Novel eIF4E-Binding Protein

**DOI:** 10.3390/genes14112076

**Published:** 2023-11-14

**Authors:** Lauren Pugsley, Sai Kiran Naineni, Mehdi Amiri, Akiko Yanagiya, Regina Cencic, Nahum Sonenberg, Jerry Pelletier

**Affiliations:** 1 Department of Biochemistry, McGill University, Montreal, QC H3G 1Y6, Canada; lauren.pugsley@mail.mcgill.ca (L.P.); sai.naineni@mail.mcgill.ca (S.K.N.); mehdi.amiri@mail.mgill.ca (M.A.); nahum.sonenberg@mcgill.ca (N.S.); 2 Arcalis, Inc., Kashiwa 277-0871, Japan; unangepasse11@gmail.com; 3 Rosalind and Morris Goodman Cancer Institute, McGill University, Montreal, QC H3A 1A3, Canada

**Keywords:** protein synthesis, translation initiation, gene expression, mRNA, cap-dependent translation, C8ORF88, eIF4E

## Abstract

Translation initiation in eukaryotes is regulated at several steps, one of which involves the availability of the cap binding protein to participate in cap-dependent protein synthesis. Binding of eIF4E to translational repressors (eIF4E-binding proteins [4E-BPs]) suppresses translation and is used by cells to link extra- and intracellular cues to protein synthetic rates. The best studied of these interactions involves repression of translation by 4E-BP1 upon inhibition of the PI3K/mTOR signaling pathway. Herein, we characterize a novel 4E-BP, C8ORF88, whose expression is predominantly restricted to early spermatids. C8ORF88:eIF4E interaction is dependent on the canonical eIF4E binding motif (4E-BM) present in other 4E-BPs. Whereas 4E-BP1:eIF4E interaction is dependent on the phosphorylation of 4E-BP1, these sites are not conserved in C8ORF88 indicating a different mode of regulation.

## 1. Introduction

Cap-dependent translation is the predominant mechanism of translation initiation in eukaryotes [1]. The cap structure, m^7^GpppN (where N is any nucleotide and m is a methyl group), links a methyl modified guanosine via a 5′-5′ triphosphate bridge to the first mRNA-transcribed nucleotide [2,3]. The activity of the cap in translation is mediated by the eukaryotic initiation factor (eIF) 4F complex, which assembles on the 5′ cap of mRNA to recruit the ribosome and its associated factors. The eIF4F complex consists of the cap-binding protein eIF4E, the DEAD-box RNA helicase eIF4A, and the large scaffolding protein eIF4G. The rate-limiting factor in eIF4F assembly is eIF4E, and as such, its availability governs rates of translation initiation [4,5,6,7,8].

eIF4E also interacts with eIF4E-binding proteins (4E-BPs). There are three 4E-BP homologs in mammals that share ~60% identity, of which the best studied is 4E-BP1 [4]. There are several 4E-BPs in yeast [9,10]. The association of eIF4E with 4E-BPs is disrupted by the serine/threonine kinase mTOR, a master regulator of several cellular processes, such as protein synthesis, cell growth and proliferation, lipid metabolism, cytoskeletal organization, mitochondrial function, and autophagy [11,12,13,14]. Activation of mTOR results in stepwise phosphorylation of several conserved residues in 4E-BPs, decreasing 4E-BP1′s affinity for eIF4E and leading to increased eIF4F levels [15]. A canonical eIF4E binding motif (4EBM) interacts with a conserved hydrophobic pocket on the dorsal surface of eIF4E via the sequence YXXXXLΦ (where Y denotes tyrosine, X is any amino acid, L denotes leucine and Φ is a hydrophobic residue), which is critical for interacting with eIF4E [16]. A second site, referred to as a non-canonical 4EBM, is present 15–30 residues downstream of the canonical 4EBM and significantly increases the affinity of the 4E-BPs for eIF4E [17,18,19,20]. Recently, it was discovered that the conservation of 4E-BPs extends to plants and is controlled by TOR [21].

Dysregulation of 4E-BP family members has been associated with disease states. Overexpression of hyperphosphorylated 4E-BP1 has been extensively linked to different adverse outcome variables across a wide variety of cancers [22,23,24,25,26,27]. 4E-BP2 is the 4E-BP isoform predominantly expressed in the brain, and in recent years, loss of 4E-BP2 has been linked to the development of autism spectrum disorder (ASD)-associated behaviours in mice [28]. Lastly, 4E-BP3 was found to be important in maintaining translational repression during prolonged periods of mTORC1 inhibition [29,30,31].

In 2017, Sonenberg and colleagues published the results of a BioID assay [32] seeking to identify novel interacting partners of eIF4E and 4EHP, the latter being an eIF4E family member sharing 28% homology with eIF4E but acting as a translational repressor [33,34,35]. Among the datasets of the BioID assay, a protein of unknown function, C8ORF88, was found to be a high-confidence binding partner of eIF4E but not 4EHP [34]. Gene Ontology (GO) analysis predicted C8ORF88 to be an eIF4E binding protein and a negative regulator of translation. In this work, we characterize C8ORF88 and validate its interaction with eIF4E.

## 2. Materials and Methods

### 2.1. Sequence Alignments

All genomic DNA sequences were obtained from the NCBI Gene database [17]. Gene Accession numbers used were as follows: C8ORF88 (NM_001190972.2), 4E-BP1 (NP_004086.1), 4E-BP2 (NP_004087.1), 4E-BP3 (NP_003723.1). Amino acid sequence of C8ORF88 was obtained using the ExPASy Translation Tool [36]. Sequence alignments of C8ORF88 with the 4E-BP homologs were performed using the Clustal Omega Multiple Sequence Alignment Tool [37].

### 2.2. Northern Blot Analysis

Mouse tissue RNA was obtained from Zyagen. RNA (4 µg) or ssRNA Ladder (New England Biolabs, NEB) was prepared to a volume of 20 µL with loading buffer (50% deionized formamide, 6% formaldehyde, 20 mM sodium borate, 0.2 mM EDTA pH 8.3). Samples were denatured at 65 °C for 2 min directly prior to loading on a 1.2% agarose formaldehyde gel (6% formaldehyde, 20 mM sodium borate, 0.2 mM EDTA pH 8.3). The gel was run at 90V for 3 h with buffer recirculation, after which the ssRNA Ladder was cut from the gel and stained using SYBR™ Gold Nucleic Acid Gel Stain (Invitrogen, Carlsbad, CA, USA) followed by ultraviolet (UV) imaging. Samples were transferred to a Hybond N+ membrane (GE Healthcare, Aurora, OH, USA) through capillary action in 10X SSC Buffer (1.5 M NaCl, 150 mM sodium citrate) at room temperature for 48 h. The membrane was UV crosslinked using a Stratagene Stratalinker 2400 at a UV power of 1200.

The DNA probe was radiolabelled with α-^32^P-dATP (Perkin Elmer, Waltham, MA, USA) using the Takara Random Primer DNA Labeling Kit Ver.2.0 as per the manufacturer’s instructions. Unincorporated α-^32^P-dATP was removed by EZ-10 DNA spin column (Bio Basic, Markham, ON, Canada). C8Orf88 probe was generated from the full-length protein coding region of the cDNA, isolated through restriction digest of plasmid pGEX-6P1-Flag-mC8Orf88. An 18S rRNA probe was generated by PCR amplification of mouse tissue cDNA generated from mouse liver RNA using M-MuLV Reverse Transcriptase (New England Biolabs, NEB) with the following primers: 18S_rRNA_For, ^5′^AACTGTGGTAATTCTAGAGC^3′^, 18S_rRNA_Rev, ^5′^CCATCGAAAGTTGATAGGGC^3′^.

The membrane was blocked with 15mL hybridization buffer (50% formamide, 1× Denhardt’s solution, 0.8 M NaCl, 4% Dextran SO_4_, 1% sodium pyrophosphate, 50 mM Tris pH 7.5, 0.5% SDS, 1mg/mL salmon sperm DNA) at 42 °C overnight in a rotating hybridization oven. Following pre-hybridization, 1 × 10^6^ cpm/mL of denatured radiolabelled probe was added directly to the hybridization buffer and incubated at 42 °C in a rotating hybridization oven for 16–24 h. The membrane was washed twice each for 30 min with decreasing concentrations of SSC buffer (2×, 1×, 0.5× and 0.2×) containing 0.1% SDS at 65 °C. Membranes were exposed at −80 °C to X-ray film for 30 min (rRNA probe) and 4 days (C8Orf88 probe).

### 2.3. RT-qPCR

Mouse tissue RNA was obtained from Zyagen. cDNA was generated from RNA (1 µg) using M-MuLV Reverse Transcriptase (New England Biolabs, NEB, Whitby, ON, Canada). cDNA was diluted 1:10 and 1 µL was used in a 10 µL qPCR reaction using SsoFast EvaGreen Supermix (Bio-Rad, Mississauga, ON, Canada) as per the manufacturer’s instructions. Raw cycle threshold (Ct) values were compared to assess expression levels.

Primers were designed using NCBI Primer-BLAST and were obtained from Integrated DNA Technologies (IDT):

mC8Orf88_qPCR_For, ^5′^TGGGTCTTGAGGCGTATG^3′^
mC8Orf88_qPCR_Rev, ^5′^ATCTGCCCACTCCACTTTGT^3′^
β-actin_qPCR_For, ^5′^TTCCTTCTTGGGTATGGAATCC^3′^
β-actin_qPCR_Rev, ^5′^AGGAGCAATGATCTTGATCTTC^3′^

### 2.4. Plasmids and Recombinant Proteins

pLeGo-Flag-C8ORF88 and pLeGo-Flag-C8ORF88(Δ6) were generated through restriction cloning of gBlocks™ Gene Fragments (Integrated DNA Technologies, Coralville, IA, USA) into the pLeGo-SP6 backbone. pLeGo-Flag-4E-BP [5A] was generated through PCR amplification of 4E-BP[5A] from pCMV-4E-BP[5A] using PCR primers containing an N-terminal Flag extension with the following primers:

Flag_4E-BP[5A]_For, ^5′^CCCCATATCATCGTGGGATCCCAATGGACTACAAGGACGACGACGATAAGATGTCGG^3′^

Flag_4E-BP[5A]_Rev, ^5′^CGAGATTTCACTGTTGAATTCTTAAAGTCCATCTCAAA^3′^

The PCR product was cloned into the pLeGo-SP6 backbone.

pGEX-C8ORF88 and pGEX-C8ORF88(Δ6) were generated through restriction cloning of gBlocks™ Gene Fragments (Integrated DNA Technologies) containing sequences codon optimized for *Escherichia coli* K12 into the pGEX-6P1 backbone. To generate recombinant GST-C8ORF88 and GST-C8ORF88(Δ6) protein, Rosetta™(DE3) Competent Cells (Sigma-Aldrich, Oakville, ON, Canada) were transformed with pGEX-C8ORF88 and pGEX-C8ORF88(Δ6) and induced with 0.5 mM IPTG for 3 h at 37 °C in a shaking incubator. A bacterial pellet was harvested via centrifugation, and a GST-based purification performed [38].

### 2.5. Cell Culture, Transfections and Lysate Preparation

HEK293T and HeLa cells were grown in Dulbecco’s modified Eagle’s medium (DMEM) supplemented with 10% bovine growth supplemented serum (BGSS), 1% penicillin-streptomycin antibiotics, and 2mM L-Glutamine at 37 °C and 5% CO_2_. Cells were transiently transfected with pLeGo-Flag-C8ORF88, pLeGo-Flag-4E-BP[5A], pLeGo-Flag-6aa-C8ORF88(Δ6), or empty pLeGo plasmid DNA in a 6-well plate (3.5 µg) or 10cm dish (10 µg). Transfections were performed using either polyethylenimine (PEI) [39] or calcium phosphate [40].

Two methods of cell lysate preparation were utilized. Firstly, for NP40 lysis cells were washed in ice cold PBS and scraped. Cells were then rinsed in PBS and lyzed in 400 µL NP40 lysis buffer (20 mM Tris pH 7.5, 150 mM NaCl, 0.5% NP40, 2mM EDTA pH 8.3) supplemented with protease inhibitors (2 µg/mL leupeptin, 10 µg/mL aprotinin, 2.5 µM pepstatin A) for 10 min on ice. Lysates were cleared of cellular debris via centrifugation. Secondly, for RIPA lysis cells were washed in ice cold PBS and scraped. Cells were rinsed in PBS and lyzed in 100 µL RIPA lysis buffer (20 mM Tris_._ pH 7.6, 100 mM NaCl, 1 mM EDTA pH 8.0, 1 mM EGTA pH 8.0, 1% NP-40, 0.5% deoxycholic acid, 0.1% SDS, 10 mM NaF, 20 mM β-glycerophosphate, 1 mM PMSF) supplemented with protease inhibitors (2 µg/mL leupeptin, 10 µg/mL aprotinin, 2.5 µM pepstatin A) for 10 min on ice. Lysates were cleared of cellular debris via centrifugation.

### 2.6. Western Blot Analysis

Samples were resolved on either a 10% or 12.5% SDS-polyacrylamide gel (PAGE) and transferred to 0.2 µm Immun-Blot™ PVDF Membrane (BIO-RAD). Membranes were blocked for 1 h in 5% milk in Tris-buffered saline supplemented with 0.2% Tween 20 (TBS-T). The primary antibodies anti-DDDDK (Abcam, Waltham, MA, USA) (recognizes FLAG-tag sequence), anti-eIF4E (Cell Signaling Technology, Danvers, MA, USA) and anti-His (Cell Signaling Technologies) were diluted 1:1000 in 5% milk in TBS-T. The primary antibody anti-GST (Santa Cruz, Dallas, TX, USA) was diluted 1:500 in 5% milk in TBS-T. Membranes were incubated with the primary antibody for 1 h at room temperature and washed 3 times with TBS-T before addition of species-appropriate horseradish peroxidase (HRP) conjugated secondary antibody (Jackson ImmunoResearch, West Grove, PA, USA) in 5% milk in TBS-T. Membranes were washed 3 times with TBS-T before addition of ECL UltraScence Western Substrate (FroggaBio, Concord, ON, Canada) and exposed to Medical X-ray Blue film (Carestream, Rochester, NY, USA).

### 2.7. In Vitro Translation and m^7^GTP Cap-Affinity Chromatography

Capped mRNAs were synthesized using SP6 RNA polymerase (New England Biolabs, NEB) from linearized templates of pLeGo-Flag-C8ORF88, pLeGo-Flag-4E-BP[5A], pLeGo-Flag-C8ORF88(Δ6) and pLeGo-Flag-eIF4E in the presence of anti-reverse cap analog (ARCA) [41] and purified using a G50 Sephadex spin column. mRNAs encoding Flag-C8ORF88, Flag-4E-BP[5A] or Flag-C8ORF88(Δ6) (0.5 µg) were translated in conjunction with eIF4E (0.5 µg) in wheat germ extract (Promega, Madison, WI, USA). Each mRNA pair was translated in a 30 µL reaction for 1 h at 25 °C in the presence of 15 µCi EasyTag™ L-[^35^S]-methionine (PerkinElmer). Translations were then subjected to cap-affinity chromatography using a γ-amino phenyl m^7^GTP (C10 spacer) agarose (Jena Bioscience, Jena, Germany). Prior to use, beads were calibrated four times with LCB Buffer (20 mM Hepes pH 7.5, 0.1 M KCl, 0.2 mM EDTA pH 8.0). Translations were incubated on the resin for 2 h at 4 °C with end-over-end rotation. The resin was washed 3 times with LCB Buffer and 2 times with 500 µM GTP before elution in 500 µM m^7^GTP. For each sample, the input, second GTP wash and m^7^GTP elution were resolved on a 12.5% SDS-PAGE which was then treated with EN^3^HANCE™ (PerkinElmer) prior to drying and exposure to X-ray film (Carestream). For cap-pulldowns of recombinant His-eIF4E (2.5 µg) and GST-C8ORF88, GST-4E-BP1 or GST-C8ORF88(Δ6) (0.5 µg), the respective proteins were incubated in 50 µL LCB Buffer at RT for 1h followed by incubation on the resin, washing and elution as above. Input and m^7^GTP elutions were resolved on a 10% SDS-PAGE and analyzed by Western blotting using anti-GST (Santa Cruz) and anti-His (Cell Signaling Technologies) antibodies.

### 2.8. GST Pulldown

GST-C8ORF88, GST-4E-BP1 or GST-C8ORF88(Δ6) (2.5 µg) were incubated with 0.5 µg His-eIF4E or His-eIF4E(W73A) in 50 µL Binding Buffer (20 mM Tris pH 7.5, 100mM KCl, 10% glycerol, 0.1% NP-40) for 1 h at room temperature. Fifty microliters of a 50% Glutathione Sepharose^®^ 4 Fast Flow (Cytiva, Vancouver, BC, Canada) slurry, that had been washed 3 times with Binding Buffer, was added to the protein mix and incubated for 1 h on ice. The beads were then washed 3 times with Binding Buffer prior to elution in 50 µL 10 mM Reduced Glutathione for 1 h on ice. Proteins were resolved on a 10% SDS-PAGE and analyzed by Western blotting using anti-GST (Santa Cruz) and anti-His (Cell Signaling Technologies) antibodies.

### 2.9. Co-Immunoprecipitation and m^7^GTP Cap-Affinity Chromatography

In co-immunoprecipitation experiments, transiently transfected HEK293T cells (10 cm dish) were lyzed in NP40 lysis buffer 24 h post-transfection and cleared of cellular debris as described above. Anti-FLAG^®^ M2 magnetic beads (Millipore Sigma, Oakville, ON, Canada) were prepared by washing twice with NP40 lysis buffer. Lysates were immunoprecipitated with the antibody-coupled beads for 1 h on ice and were periodically agitated. After immunoprecipitation, beads were washed 5 times with NP40 lysis buffer and resuspended in 40 µL 1X SDS loading buffer. Samples were resolved on a 12.5% SDS-PAGE and analyzed by Western blotting using anti-DDDDK (Abcam) and anti-eIF4E (Cell Signaling Technology) antibodies.

For m^7^GTP cap-affinity chromatography, transfected HEK293T cells (10 cm dish) were lyzed in 400 µL NP40 lysis buffer 24 h post-transfection as described above. Lysates were subjected to cap-affinity chromatography using a γ-amino phenyl m^7^GTP (C10 spacer) agarose (Jena Bioscience), as described above. Samples were resolved on a 12.5% SDS-PAGE and analyzed by Western blotting using anti-DDDDK (Abcam) (recognizes FLAG-tag sequence) and anti-eIF4E (Cell Signaling Technology) antibodies.

### 2.10. L-[^35^S]-Methionine/Cysteine Labelled Protein Incorporation in Cells

HeLa cells were transiently transfected to express Flag-C8ORF88, Flag-C8ORF88(Δ6) or empty vector in a 6-well plate. Twenty-four hours post-transfection, cells were re-seeded in a 24-well plate, and 48 h post-transfection the media was changed to DMEM supplemented with 10% dialyzed FBS for 1 h. For the last 15 min, 150–225 μCi/mL of LLC EasyTag™ EXPRESS [^35^S]-methionine/cysteine Protein Labeling Mix (Perkin Elmer, Waltham, MA, USA) was added to each well. At the end of the incubation, media was removed, and cells were rinsed with cold PBS. Cells were lyzed in 40 µL of RIPA lysis buffer at 4 °C. Lysates were TCA precipitated on Whatman paper that had been pre-blocked with MEM Amino Acids Solution (50X) without methionine (Gibco/Fisher Scientific, Ottawa, ON, Canada). Radioactivity was measured via scintillation counting.

## 3. Results

### 3.1. Sequence Alignment Reveals Homology between C8ORF88 and the 4E-BP Homologs

**Figure 1:**
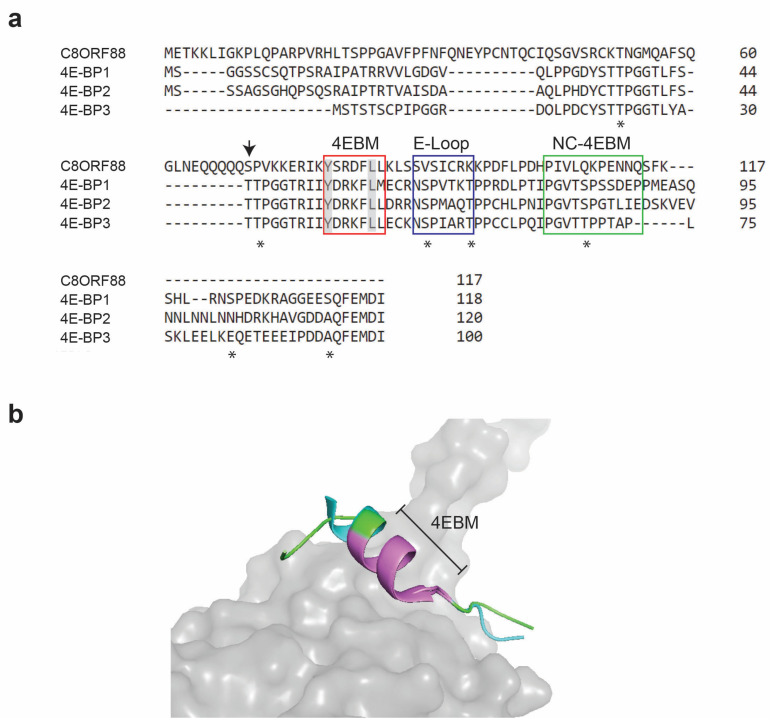
C8ORF88 amino acid sequence alignment with 4E-BP homologs. (**a**) Conserved sequences in C8ORF88 and 4E-BPs implicated with eIF4E binding activity are identified with boxes and include the canonical 4EBM (C-4EBM) (red), the elbow loop domain (E-loop) (blue) and the non-canonical 4EBM (NC-4EBM) (green). The conserved tyrosine (Y) and leucine (L) of the canonical 4EBM sequence YXXXXLΦ are highlighted in grey. Sequence alignment was performed using the Clustal Omega Multiple Sequence Alignment Tool. Asterisks underneath the sequence denote phosphorylation sites previously identified in 4E-BP1 [15]. Downward arrow denotes C8ORF88 S70, which according to PhosphoSitePlus (https://www.phosphosite.org/proteinAction.action?id=35695900&showAllSites=true, accessed on 15 February 2022) represents a single minor phosphorylation site. (**b**) Superimposition of 4E-BP1 and C8ORF88. Predicted structure of C8ORF88 flanking 4EBM (https://alphafold.ebi.ac.uk/entry/P0DMB2, accessed on 20 July 2023) was superimposed on 4E-BP1:eIF4E crystal structure (PDB 1WKW) using PyMOL. C8ORF88 is in cyan, 4E-BP1 in green and eIF4E in grey. The canonical 4EBM is colored violet.

*C8ORF88* encodes a polypeptide of 117 amino acids (Figure 1a). Sequence alignment of C8ORF88 with 4E-BP revealed ~20–26% identity to the 4E-BP homologs and conservation of the 4EBM (Figure 1a). Three-dimensional structural alignment indicated that the C8ORF88 4EBM superimposed well onto the corresponding domain of eIF4E-bound 4E-BP1 (Figure 1b). Of the seven 4E-BP1 phosphorylation sites, two amino acids are also present in C8ORF88 (Figure 1a). However, analysis of the PhosphoSitePlus database indicated that neither of these are phosphorylated, but rather there is a minor site used at S70 (Figure 1a, downward arrow). Superimposition of the AlphaFold predicted C8ORF88 structure with 4E-BP1 in the eIF4E:4E-BP1 crystal structure showed an excellent fit across the 4EBM (Figure 1b).

### 3.2. Expression Profile of C8Orf88

**Figure 2:**
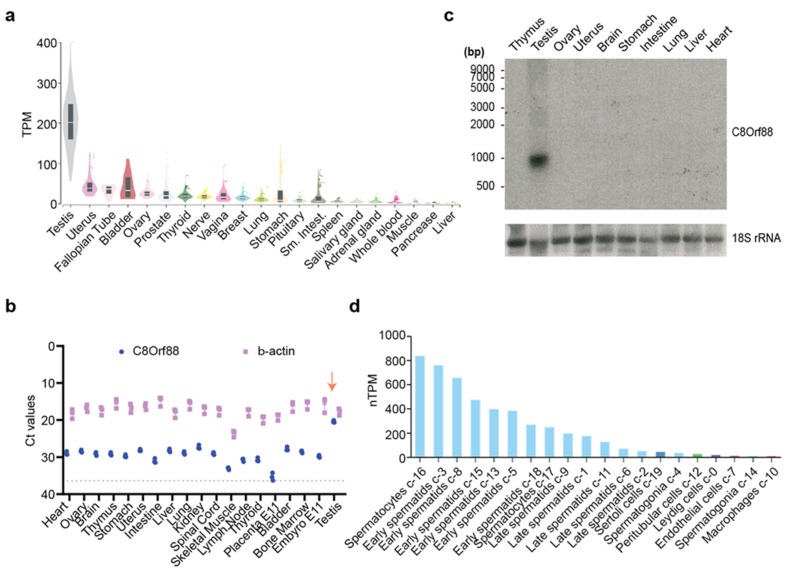
Tissue expression pattern of C8ORF88. (**a**) Bulk tissue C8ORF88 expression from GTEx Portal (https://www.gtexportal.org/home/gene/C8ORF88, accessed on 15 July 2023). (**b**) Ct values obtained from *C8Orf88* and *β-actin* amplification using RT-qPCR. The dotted line represents the Ct value of the background signal from *β-actin*’s blank control. The downward arrow identifies highest expression levels of *C8Orf88* in testes. (**c**) Northern blot analysis of *C8Orf88* mRNA from mouse tissues. (**d**) Single cell data showing C8ORF88 expression levels in testis as obtained from The Human Protein Atlas (https://www.proteinatlas.org/ENSG00000253250-C8orf88/single+cell+type, accessed on 15 July 2023).

Perusal of the GTEx database portal shows that C8ORF88 expression was the highest in testis, with very little expression in other tissues (Figure 2a). This contrasts to levels of 4E-BP1 which are consistently higher across a broad tissue set (Figure S1a). These data were verified by RT-qPCR analysis of *C8Orf88* mRNA levels across several tissue types (Figure 2b). Lastly, Northern blotting analysis indicated the presence of a single ~900 bp mRNA present in testis RNA (Figure 2c). RNA-Seq data derived from human tissue samples available through The Human Protein Atlas [42] maps expression of C8ORF88 to germ cells of testes. More specifically, RNA expression was highest in spermatocytes and early spermatids and diminished as they matured (Figure 2d). This is strikingly different from 4E-BP1 expression where expression was highest in late spermatids (Figure S1).

### 3.3. In Vitro Association between C8ORF88 and eIF4E

**Figure 3:**
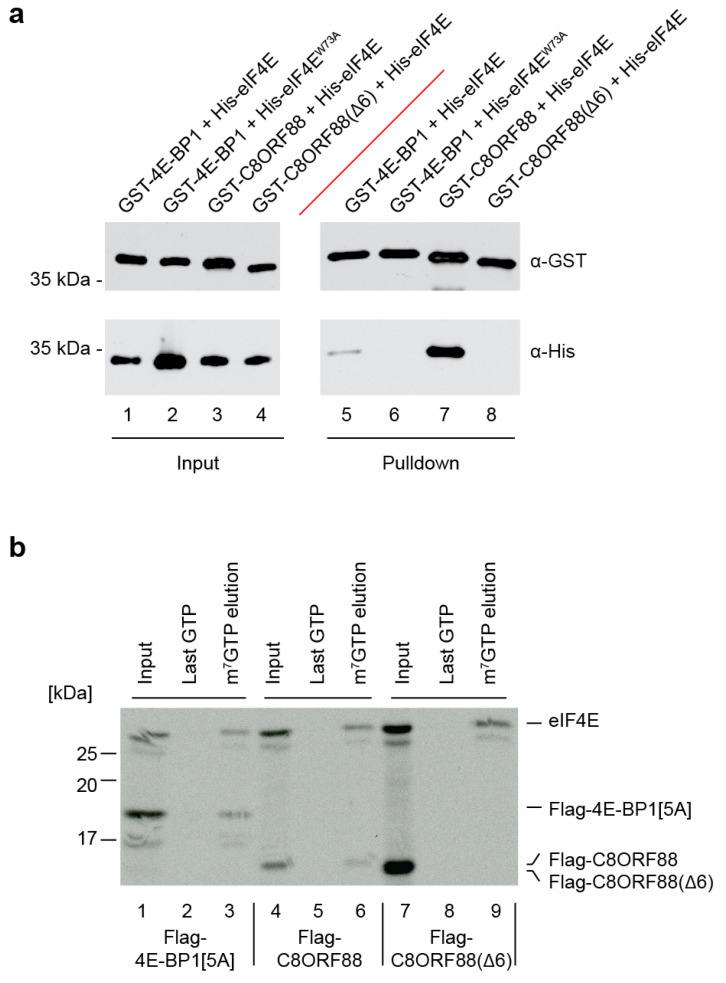
In vitro association between C8ORF88 and eIF4E. (**a**) Pulldown of GST-tagged C8ORF88, 4E-BP1 and C8ORF88(Δ6) recombinant proteins in combination with His-4E or His-4E^W73A^. (**b**) Recombinant proteins were generated by in vitro translations in wheat germ extracts in the presence of [^35^S]-labelled methionine. Reactions were then incubated with m^7^GTP affinity resin. After binding, the resin was washed three times with LCB buffer and twice with 500 µM GTP before eluting with 500 µM m^7^GTP. For each reaction, the input, last GTP wash and m^7^GTP elution were resolved by SDS-PAGE, EN^3^HANCED, and visualized using autoradiography.

To investigate the interaction between C8ORF88 and eIF4E, recombinant tagged proteins were purified and used in GST pulldown assays (Figure 3a). Here, GST-4E-BP1 was used as a positive control as it has previously been shown to interact with eIF4E in this assay. In addition to wild-type C8ORF88, we also expressed a mutant devoid of the canonical 4EBM by deletion of six amino acids YSRDFL (Δ6). Another control used was His-eIF4E^W73A^, which harbors a mutation in the conserved Trp73 on the dorsal surface of eIF4E and is essential for interaction with the canonical 4EBM [43]. Using a Glutathione Sepharose solid support matrix, all GST-tagged proteins (4E-BP1 and C8ORF88) were recovered (Figure 3a, lanes 5–8). His-eIF4E, but not His-eIF4E^W73A^, was detected in the pulldown fractions with GST-4EBP1, as expected. C8ORF88, but not C8ORF88(Δ6), was able to pulldown eIF4E (Figure 3a).

We next sought to determine whether binding of C8ORF88 to eIF4E would compromise eIF4E:cap interaction. To this end, we produced radiolabeled recombinant proteins in wheat germ extracts and undertook eIF4E pull down experiments using an m^7^GTP affinity matrix (Figure 3b). For these experiments, we used a mutant of 4E-BP1, Flag-4E-BP1[5A]. Flag-4E-BP1[5A] harbors alanine substitutions at five phosphorylation sites (T37, T46, S65, T70 and S83) and robustly interacts with eIF4E [43,44]. From extracts containing both eIF4E and Flag-4E-BP1[5A], both proteins were clearly present in the m^7^GTP elutions (Figure 3b, compare lane 3 to 2). Affinity chromatography from extracts harboring both eIF4E and C8ORF88, revealed that C8ORF88 was associated with cap-bound eIF4E (Figure 3b, compare lanes 6 to 5). In contrast, Flag-C8ORF88(Δ6) did not interact with cap-bound eIF4E (Figure 3b, compare lane 9 to 6). This was also validated utilizing the recombinant proteins tested in Figure 3a, as a reciprocal pulldown experiment performed using an m^7^GTP affinity column instead of Glutathione Sepharose solid support matrix (Figure S2). GST-4EBP1 and GST-C8ORF88 were detected in pulldown fractions with His-eIF4E, whereas His-eIF4E was not able to pulldown GST-C8ORF88(Δ6) (Figure S2).

These results indicate that C8ORF88 can interact with free and cap-bound eIF4E in vitro, and that the 4EBM is essential for this interaction.

### 3.4. C8ORF88 Interacts with Endogenous eIF4E in a Cultured Cell Line

**Figure 4:**
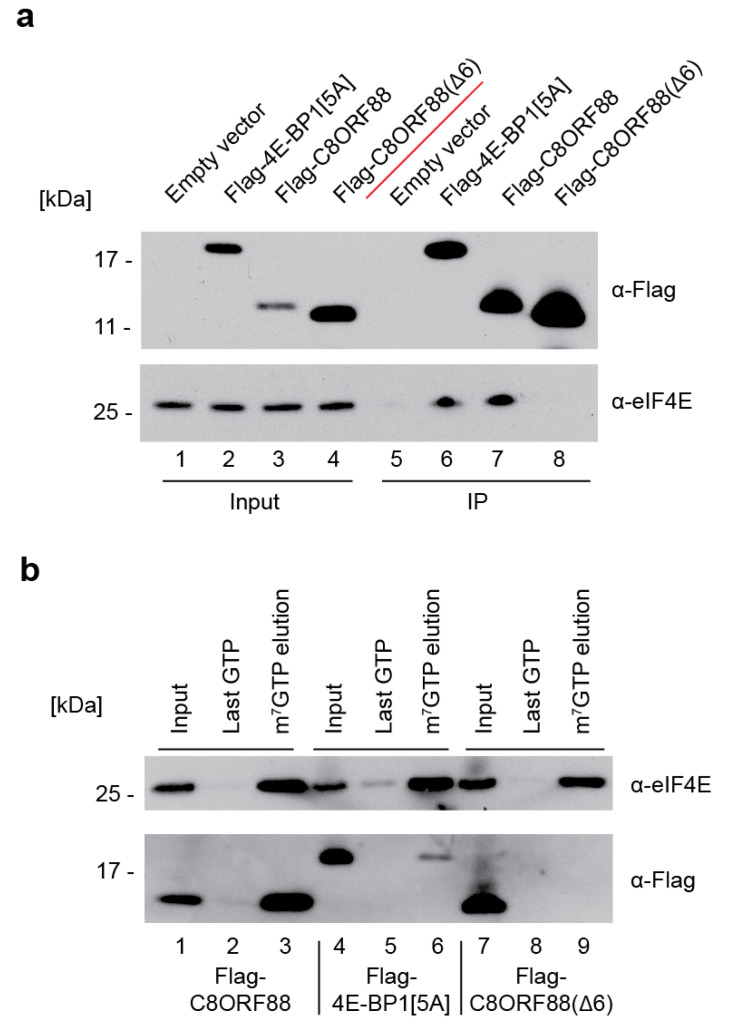
Interaction between C8ORF88 and eIF4E in cell culture. (**a**) Immunoprecipitation of transfected HEK293T cell lysates with anti-Flag antibody conjugated magnetic beads. Samples were resolved by SDS-PAGE and analyzed by Western blotting. (**b**) m^7^GTP cap-affinity chromatography of HEK293T cell lysates. The resin was washed three times with LCB buffer and twice with 500 µM GTP before elution in 500 µM m^7^GTP. For each sample the input, last GTP wash and m^7^GTP elution were resolved by SDS-PAGE and analyzed by Western blotting.

To further investigate the interaction between C8ORF88 and eIF4E, the interaction was studied in a cultured cell line. HEK293T cells were transfected to express either Flag-4E-BP1[5A], Flag-C8ORF88 or Flag-C8ORF88(Δ6), and cell lysates were harvested 24 h later. Lysates were subjected to immunoprecipitation using anti-Flag conjugated magnetic beads (Figure 4a). Endogenous eIF4E co-immunoprecipitated with Flag-4E-BP1[5A] and Flag-C8ORF88 (Figure 4a, compare lanes 6 and 7 to 5). No interaction was observed in cells expressing Flag-C8ORF88(Δ6) (Figure 4a, lane 8).

Lysates prepared from HEK293T cells transfected to express the same three Flag-tagged proteins were also subjected to cap-affinity chromatography (Figure 4b). eIF4E was eluted from the cap column for all samples, indicating it successfully bound to the resin (Figure 4b, lanes 3, 6 and 9). C8ORF88 and Flag-4E-BP1[5A] eluted from the resin with endogenous eIF4E, as expected (Figure 4b, lanes 3 and 6). Flag-C8ORF88(Δ6) was not retained on the cap column, underscoring the importance of the canonical 4EBM for eIF4E interaction (Figure 4b, lane 9). Together, these data confirm the interaction between C8ORF88 and both free and cap-bound eIF4E.

### 3.5. Ectopic Expression of C8ORF88 Inhibits Translation in a Cultured Cell Line

**Figure 5:**
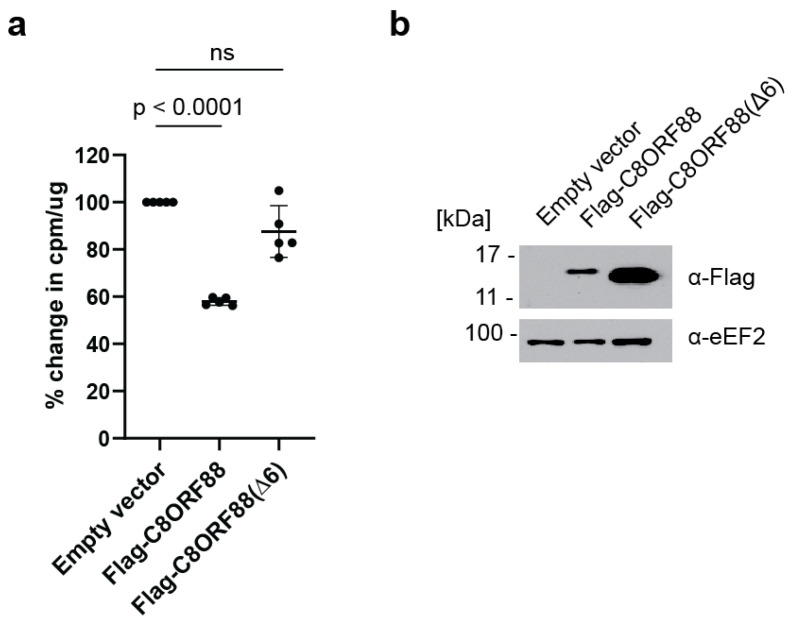
Overexpression of C8ORF88 leads to a reduction in global translation rates. (**a**) HeLa cells transfected to overexpress Flag-C8ORF88, Flag-C8ORF88(Δ6) or empty vector, were treated with [^35^S]-labelled protein mix for 1 h, 48 h after transfection. Incorporation of [^35^S]-labelled proteins was assessed via scintillation counting and standardized to the empty vector control. *p* values were determined using Dunnett’s multiple comparison test. (**b**) Expression of Flag-tagged proteins confirmed by Western blotting.

HeLa cells were transiently transfected to express Flag-C8ORF88 or Flag-C8ORF88(Δ6). Forty-eight hours post-transfection, cells were treated with [^35^S]-labelled Met/Cys for 1 h, lyzed, and radiolabel incorporation was measured via scintillation counting (Figure 5). A significant reduction (42%) in global translation rates was observed on Flag-C8ORF88 expressing cells, compared to control cells (Figure 5a). No significant difference in translation was observed between cells expressing Flag-C8ORF88(Δ6) or receiving empty vector (Figure 5a). Expression of the indicated Flag-tagged proteins was confirmed via Western blotting (Figure 5b). These data indicate that C8ORF88 is a negative regulator of translation.

## 4. Discussion

In this study, we provide an initial characterization of a novel testes-specific eIF4E binding protein. We found that expression of C8Orf88 was highest in testis, and this was congruent with available RNA-Seq data (Figure 2) [45,46]. The highest levels of C8ORF88 expression occur early in spermatogenesis (Figure 2d), in contrast to 4E-BP1 (Figure S1b), which is highest in late spermatids. Translational control during spermatogenesis plays critical roles in stem cell maintenance, meiotic entry, completion of meiosis, and gamete differentiation [47,48]. More specifically, recruitment of selective messenger ribonucleoproteins (mRNPs) to ribosomes by eIF4 factors is recognized to play a vital role in translational control during oocyte and embryonic development [49,50]. During the development of oocytes and embryos, mRNP granules control gene expression through precise, special-temporal regulation of mRNA expression [51,52]. This careful regulation is essential for the correct progression of the developmental program [51,53]. It was hypothesized that eIF4G dissociates 4E-BPs from repressed mRNPs to activate their translation, driving progression through the stages of development [54]. Furthermore, C8ORF88 is conserved across chordates [55,56,57]. The tissue-specific expression pattern, in addition to its conservation, suggests that C8ORF88 has a physiologically significant role in testes [58,59]. The identification of C8ORF88 as a novel germ cell-specific 4E-BP may thus have important implications for translational control during spermatogenesis and development.

Testicular cancer is the most common cancer in men aged 14–44 [60]. Cancers with germ-cell origins, such as testicular seminomas, comprise ~50% of all testicular cancer cases [61]. Given the extensively characterized role of 4E-BP1 dysregulation across many malignancies [22,62,63,64,65], and the mapping of C8ORF88 expression to the germ cells of testes, C8ORF88 might play a role in tumorigenesis and disease progression in cancers of germ cell origin. In a recent whole-exome and transcriptome sequence study of germ cell tumors (GCTs), p53 was found to be expressed in all samples sequenced [66]. This is a unique and unusual feature, as around half of other solid tumor types harbor mutations in p53 [67,68]. Expression of p53 is thought to be essential for cisplatin-induced apoptosis in GCTs [69,70]. 4E-BP activity has previously been linked to p53 expression in primary fibroblasts, where knock-down of 4E-BP1 and 4E-BP2 in conjunction with p53 expression led to resistance to oncogene-driven transformation and premature senescence [24]. As C8ORF88 is a germ cell-specific 4EBP, it may play a role in senescence and apoptosis as governed by p53 in GCTs.

We validated the interaction of C8ORF88 with eIF4E both in vitro and in cell culture (Figure 3 and Figure 4). Results obtained with C8ORF88(Δ6) highlighted the importance of the 4EBM domain for this interaction, whose function in turn was associated with inhibition of translation (Figure 5). Pull-down experiments using m^7^GTP affinity resin indicated that the C8ORF88-eIF4E interaction does not preclude eIF4E binding to the cap structure. The interaction between cap-bound eIF4E and C8ORF88 is of particular interest as it shows C8ORF88 to be a competitor with other endogenously expressed 4E-BPs. Binding of 4E-BP1 to eIF4E is highly regulated by mTORC1 and occurs through hierarchical phosphorylation of first Thr^37^ and Thr^46^ and, subsequently, Ser^65^, Thr^70^, and Ser^83^ [15] (Figure 1a, denoted by asterisks). We note that none of these phosphorylation sites are present in C8ORF88, precluding regulation of eIF4E binding by mTOR in a manner analogous to 4E-BP1:eIF4E. There has been one phosphorylation site identified in C8ORF88 (Ser^70^) (Figure 1a), the significance of which has yet to be determined and suggests an additional mechanism of regulation. Additionally, different RNA expression profiles were observed for C8ORF88 and 4E-BP1, where C8ORF88 was found to have greater expression in spermatocytes and early spermatids, while 4E-BP1 was found to have greater expression in late spermatids. This raises the question of whether there is differential translational control in the early and late stages of spermatids by C8ORF88 and 4E-BP1, which is yet to be determined. The data presented here, in conjunction with the recognized importance of 4E-BPs in translational control, provides rationale for further investigation into the physiological role of this protein.

## Data Availability

Publicly available datasets were analyzed in this study. This data can be found here: PhosphositePlus (https://www.phosphosite.org/proteinAction.action?id=35695900&showAllSites=true, accessed on 15 February 2022), AlphaFold (https://alphafold.ebi.ac.uk/entry/P0DMB2, accessed on 20 July 2023), GTEx Portal (https://www.gtexportal.org/home/gene/C8ORF88, accessed on 15 July 2023) and (https://gtexportal.org/home/gene/EIF4EBP1, accessed on 15 July 2023), The Human Protein Atlas (https://www.proteinatlas.org/ENSG00000253250-C8orf88/single+cell+type, accessed on 15 July 2023) and (https://www.proteinatlas.org/ENSG00000187840-EIF4EBP1/single+cell+type, accessed on 15 July 2023).

## Supplementary Materials

The following supporting information can be downloaded at: https://www.mdpi.com/article/10.3390/genes14112076/s1, Figure S1: Protein expression levels of 4E-BP1; Figure S2: In vitro association between C8ORF88 and eIF4E.
