# Supplementary figures and images for: C8ORF88: A Novel eIF4E-Binding Protein
Source: Genes (Basel). 2023 Nov 14;14(11):2076. doi: 10.3390/genes14112076 (PMC10670996; doi:10.3390/genes14112076)

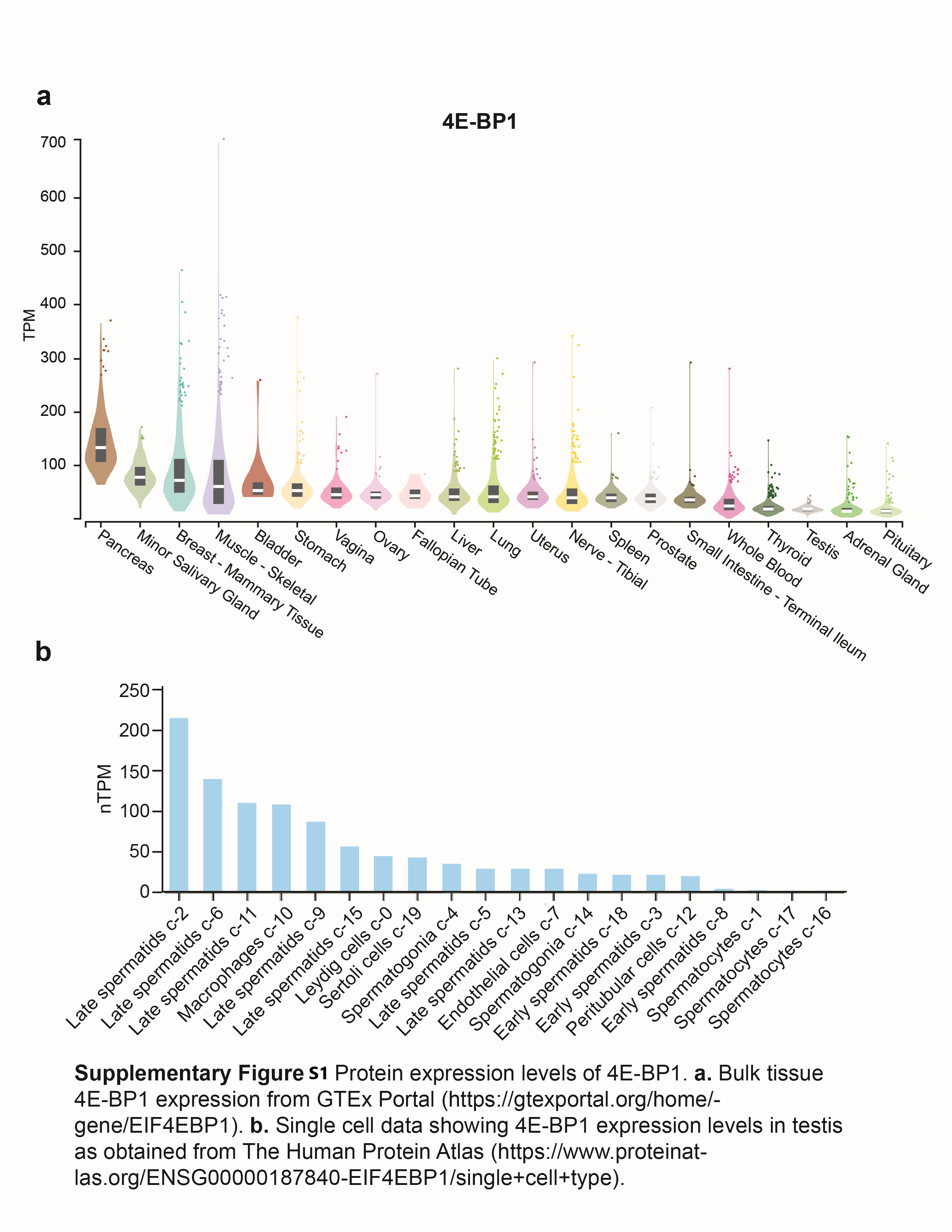

Supplement: Supplementary file 1 [file genes-14-02076-s001.zip › Supplmentary Figure S1.tif]

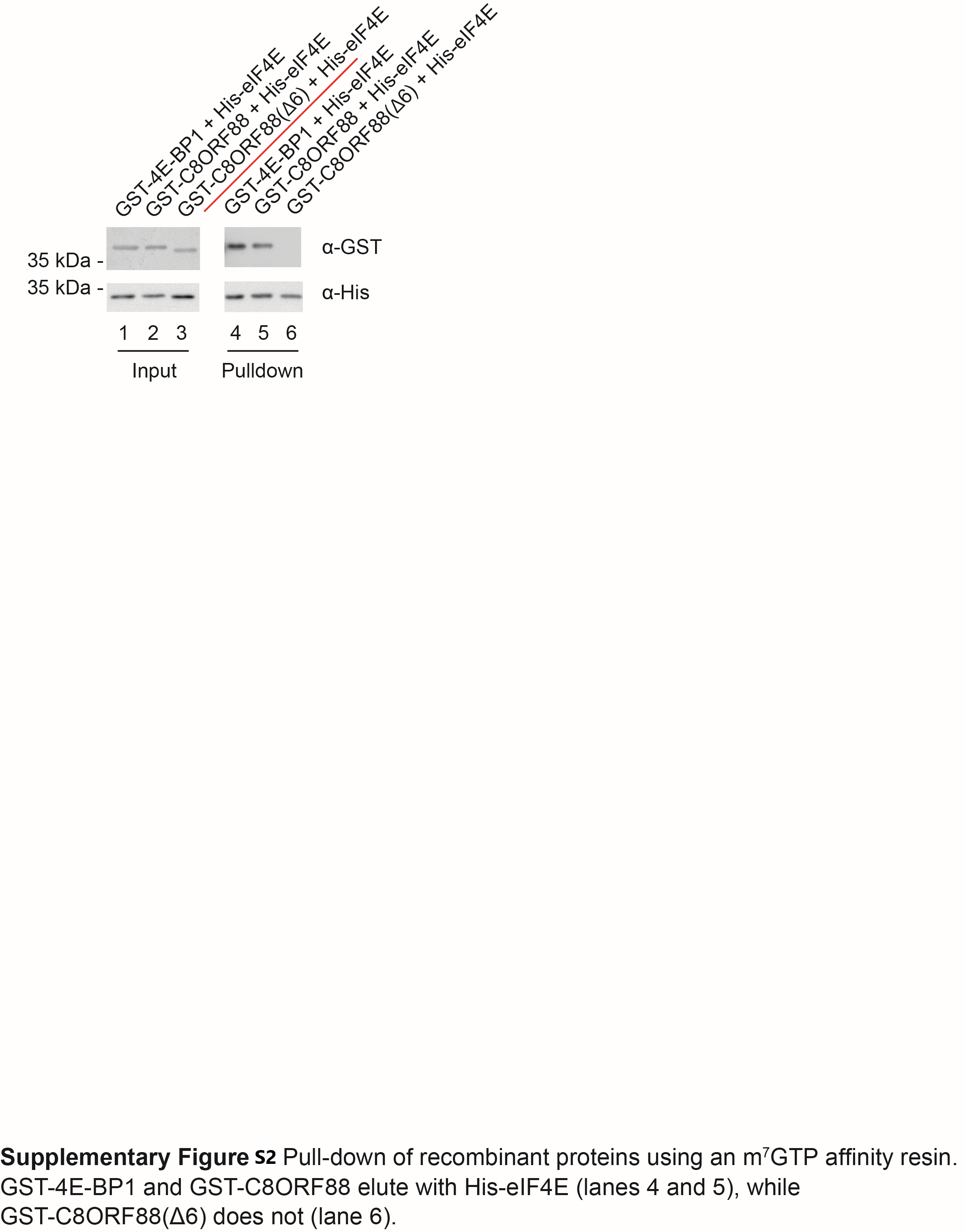

Supplement: Supplementary file 1 [file genes-14-02076-s001.zip › Supplmentary Figure S2.tif]
